# Comparative analysis of multiple inducible phages from *Mannheimia haemolytica*

**DOI:** 10.1186/s12866-015-0494-5

**Published:** 2015-08-30

**Authors:** Yan D. Niu, Shaun R. Cook, Jiaying Wang, Cassidy L. Klima, Yu-hung Hsu, Andrew M. Kropinski, Dann Turner, Tim A. McAllister

**Affiliations:** Lethbridge Research Centre, Agriculture and Agri-Food Canada, Lethbridge, AB T1J 4B1 Canada; Alberta Agriculture and Rural Development, Agriculture Centre, Lethbridge, AB T1J 4V6 Canada; College of Veterinary Medicine, South China Agricultural University, Guangdong, 510642 People’s Republic of China; Department of Biological Sciences, University of Lethbridge, Lethbridge, AB T1K 3M4 Canada; Public Health Agency of Canada, Laboratory for Foodborne Zoonoses, Guelph, ON N1G 3W4 Canada; Department of Molecular Biology, Cellular Biology and Pathobiology, University of Guelph, Guelph, ON N1G 2W1 Canada; Centre for Research in Biosciences, Department of Applied Sciences, University of the West of England, Coldharbour Lane, Bristol, BS16 1QY UK

## Abstract

**Background:**

*Mannheimia haemolytica* is a commensal bacterium that resides in the upper respiratory tract of cattle that can play a role in bovine respiratory disease. Prophages are common in the *M. haemolytica* genome and contribute significantly to host diversity. The objective of this research was to undertake comparative genomic analysis of phages induced from strains of *M. haemolytica* serotype A1 (535A and 2256A), A2 (587A and 1127A) and A6 (1152A and 3927A).

**Results:**

Overall, four P2-like (535AP1, 587AP1, 1127AP1 and 2256AP1; genomes: 34.9–35.7 kb; G+C content: 41.5–42.1 %; genes: 51–53 coding sequences, CDSs), four λ-like (535AP2, 587AP2, 1152AP2 and 3927AP1; genomes: 48.6–52.1 kb; 41.1–41.4 % mol G+C; genes: 77–83 CDSs and 2 tRNAs) and one Mu-like (3927AP2; genome: 33.8 kb; 43.1 % mol G+C; encoding 50 CDSs) phages were identified. All P2-like phages are collinear with the temperate phage φMhaA1-PHL101 with 535AP1, 2256AP1 and 1152AP1 being most closely related, followed by 587AP1 and 1127AP1. Lambdoid phages are not collinear with any other known λ-type phages, with 587AP2 being distinct from 535AP2, 3927AP1 and 1152AP2. All λ-like phages contain genes encoding a toxin-antitoxin (TA) system and cell-associated haemolysin XhlA. The Mu-like phage induced from 3927A is closely related to the phage remnant φMhaMu2 from *M. haemolytica* PHL21, with similar Mu-like phages existing in the genomes of *M. haemolytica* 535A and 587A.

**Conclusions:**

This is among the first reports of both λ- and Mu-type phages being induced from *M. haemolytica*. Compared to phages induced from commensal strains of *M. haemolytica* serotype A2, those induced from the more virulent A1 and A6 serotypes are more closely related. Moreover, when P2-, λ- and Mu-like phages co-existed in the *M. haemolytica* genome, only P2- and λ-like phages were detected upon induction, suggesting that Mu-type phages may be more resistant to induction. Toxin-antitoxin gene cassettes in λ-like phages may contribute to their genomic persistence or the establishment of persister subpopulations of *M. haemolytica*. Further work is required to determine if the cell-associated haemolysin XhlA encoded by λ-like phages contributes to the pathogenicity and ecological fitness of *M. haemolytica*.

**Electronic supplementary material:**

The online version of this article (doi:10.1186/s12866-015-0494-5) contains supplementary material, which is available to authorized users.

## Background

*Mannheimia haemolytica* is a primary etiological agent of bovine respiratory disease (BRD) [1] and a member of the *Pasteurellaceae* family which includes other zoonotic pathogens of the genera *Pasteurella*, *Haemophilus* and *Actinobacillus* [2]. *M. haemolytica* resides as a commensal bacterium in the upper respiratory tract of healthy cattle, but under some circumstances pathogenic populations can predominate [3]. Of the 12 capsular serotypes, A2 is most frequently isolated from healthy cattle, while A1 and A6 are more common in cattle with BRD [1]. The shift from a commensal to pathogenic population is a multi-factorial response to altered host conditions [3], and is likely influenced by the ecology of the microbial community, including the prevalence and nature of bacteriophages.

Bacteriophages (phages) are viruses that infect bacteria in various ecosystems including soil, water and within the intestinal tracts of animals. Based on their life cycle, phage can be classified as lytic or temperate. Upon infection, lytic phages lyse their host and release progeny viral particles that can continue the cycle of infection. In contrast, temperate phages may enter a lysogenic cycle, whereby their genomes are repressed and integrated into the bacterial chromosome. Lysogeny is common in bacteria [4] with integrated viral DNA, termed prophages (cryptic prophages or prophages remnant) being identified in almost all sequenced bacterial genomes. These genetic elements are thought to be important contributors to bacterial diversity and evolution [5, 6]. Prophages can contain genes encoding for virulence factors (e.g. toxins) that play an important role in bacterial pathogenesis. Prophages have also been shown to contribute to host survival [5] by conferring fitness against antimicrobials and other environmental selective pressures [7].

The genome of *M. haemolytica* genome is approximately 2.5–2.7 Mb [8–11]. *In*-*silicon* PHAST analysis [12] of eight sequenced strains revealed that they carry between 4 and 10 prophages about half of which are deemed to be intact. Genomic analysis of *M. haemolytica* serotype A1 PHL213 (GenBank accession #: AASA00000000) and *M. haemolytica* serotype A2 str. OVINE (GenBank accession #: ACZX00000000) revealed that phage associated genes accounted for up to 30 % of the unique genes within their genomes [10]. Previously, we found that prophages are widespread within the genome of *M. haemolytica* and contribute significantly to host diversity [13]. The objective of the current study was to conduct a comparative genomic analysis of temperate phages induced from *M. haemolytica* strains representing serotypes A1, A2 and A6.

## Results and discussion

### Induction growth curve

**Fig. 1:**
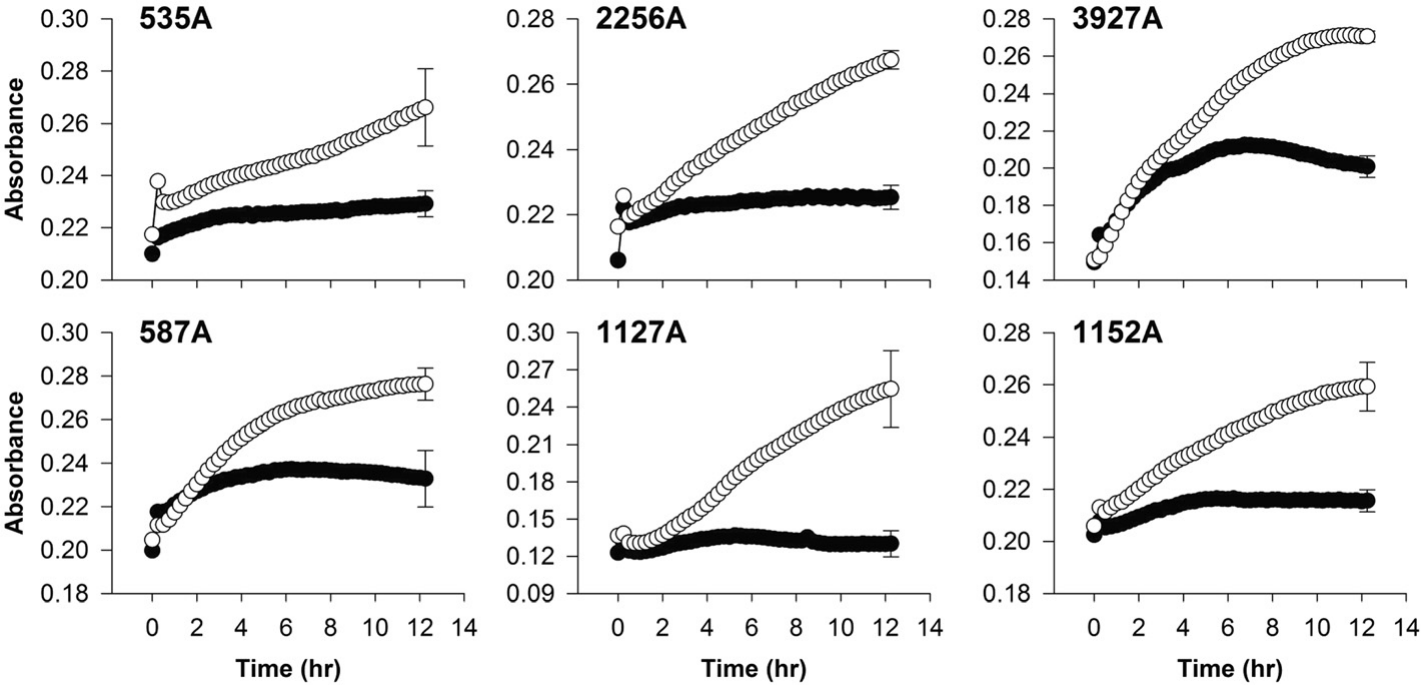
Absorbance of dual wavelength (450–600 nm) measures of log-phase *Mannheimia haemolytica* strains. Strains were induced at 0 h with mitomycin C (●) or untreated (○). Values are means with average SD *error bar* on the final point, *n* = 7

Growth curves of induced *M. haemolytica* strains all showed an obvious depreciation of growth compared to equivalent cultures not treated with mitomycin C (Fig. 1). No spontaneous release of prophages was observed from any of the *M. haemolytica* strains.

### Genomic features

P2- and λ-like phages were induced from all *M. haemolytica* strains with the exception of strain 3927A, which released λ-and Mu-like phages. Genomes were sequenced, assembled and annotated resulting in four P2-, four λ- and one Mu-like phages (Table 1). Annotation of the genomes is shown in Tables 2 and 3 and additional files (Tables S1−S9).

**Table 1:** Genomic nature of the temperate phages induced from *Mannheimia haemolytica*

Strains	Serotypes	# phages induced	Designation	Taxonomy	Genome size (bp)	Total CDSs	G+C content (%)	Reference
535A	1	2	535AP1	P2, *Myoviridae*	34,565	51	41.6	This study
			535AP2	λ, *Siphoviridae*	50,078	79	41.3	This study
2256A	1	2	2256AP1	P2, *Myoviridae*	34,926	52	41.5	This study
587A	2	2	587AP1	P2, *Myoviridae*	35,764	51	42.1	This study
			587AP2	λ, *Siphoviridae*	48,594	77	41.3	This study
1127A	2	2	1127AP1	P2, *Myoviridae*	36,745	52	42.0	This study
1152A	6	2	1152AP1	P2, *Myoviridae*	34,719	53	41.6	[13]
			1152AP2	λ, *Siphoviridae*	52,139	79	41.1	This study
3927A	6	2	3927AP1	λ, *Siphoviridae*	52,049	83	41.4	This study
			3927AP2	Mu, *Myoviridae*	33,755	50	43.1	This study

**Table 2:** Major gene products shared among lambda-like phages induced from *Mannheimia haemolytica*

No. (Gene name)	Size (aa)/MW (kDa)/pI	Function	Motifs^a^	Best homologs	% identity (aa) to Aaphi23^b^	Absent in phages
1 *terS*	174/19.1/5.4−5.6	Terminase, small subunit	Terminase_2 (pfam03592)	Bacteriophage terminase small subunit (*M. haemolytica*)	52−54	
2 *terL*	406−410/46.3−47.3/5.9−6.1	Terminase, large subunit	Terminase_3 superfamily (cl12054)	Phage terminase, large subunit, PBSX (*M. haemolytica*)	19−21	
3 *porT*	458−467/50.6−52.2/4.9−5.1	Portal protein	phage_prot_Gp6 (pfam05133)	Hypothetical protein (*M. haemolytica*)	20−25	
4 *MHP*	312−553/35.4−63.3/9.3−9.5	Head morphogenesis protein	Phage_Mu_F superfamily (cl10072)	Hypothetical protein (*M. haemolytica*)	12−23	
5 *MTP*	160/17.3/5.1	Major tail protein	Phage_tail_2 (pfam06199)	Phage major tail protein (*M. haemolytica*)		1152AP2
6	137/15.1/10.2	Hypothetical protein	DUF4128 (pfam13554)	Hypothetical protein (*M. haemolytica*)		535AP2, 3927AP1, 587AP2
7 *hicB*	138/15.5/4.6	HicB	UPF0150 (pfam03681)	Toxin-antitoxin , antitoxin component, HicB (*M. haemolytica*)		1152AP2
8 *hicA*	58/6.6/10.1	HicA	YcfA (pfam07927)	Toxin-antitoxin, toxin component, HicA (*M. haemolytica*)		1152AP2
9 *TMP*	816−1008/88.1−108.8/5.2−6.2	Tail length tape measure protein	Tape_meas_nterm superfamily (cl15680); TMD(1)	Tail length tape measure-related protein (*M. haemolytica*)	17−19	
10 *M*	107−109/12.3−12.5/8.8−9	Minor tail protein M	Phage_min_tail (pfam05939)	Gifsy-1 prophage VmtM (*M. haemolytica*)		
11 *L*	156/17.6/5	Minor tail protein L	Phage_tail_L superfamily (cl01908)	Phage-related minor tail protein L (*M. haemolytica*)		1152AP2
12 *L*	238/26.5/6−6.3	Minor tail protein L	Phage_tail_L superfamily (cl01908)	Phage minor tail protein L (*M. haemolytica*)		
13 *K*	243/28.5/5.3	Minor tail protein K	MPN_NLPC_P60 (cd08073)	Bacteriophage tail protein (*M. haemolytica*)		
14 *I*	196−209/20.9−22.1/9.6−9.9	Tail assembly protein I	Lambda_tail_I superfamily (cl01945); TM(2)	Bacteriophage tail protein and tail assembly protein I (*M. haemolytica*)	15−19	
15 *J*	1954−2352/213.3−255/6.3−8	Host specificity protein J	Phage-tail_3 (pfam13550); TM(2)	Host specificity protein J (*M. haemolytica*)		
16 *int*	329−351/37.9−40.8/9.7−9.9	Integrase	Phage_integrase (pfam00589); Phage_integ_N superfamily (cl07565); DNA_BRE_C superfamily (cl00213)	Integrase/recombinase (*M. haemolytica*)	17−24	
17 *MTase*	163/19.2/8.3	Methyltransferease	Methyltransf_25 (pfam13649)	Putative bacteriophage methyltransferase (*M. haemolytica*)		3927AP1, 1152AP2, 587AP2
18	127/14.6/5.6	Hypothetical protein	flap endonuclease-1-like (cl14815)	Hypothetical protein (*M. haemolytica*)		535AP2, 587AP2
19 *ant*	218−233/26−26.9/7.1−7.7	Antirepressor protein Ant	P22_AR_N (pfam10547); P22_AR_C superfamily (cl11179); AntA superfamily (cl01430)	Antirepressor protein Ant (*M. haemolytica*, *Avibacterium paragallinarum* for 587AP2)	19−21	535AP2
20	71/8/9.9	Hypothetical protein	PRK11675 superfamily (cl08198)	Hypothetical protein (*M. haemolytica*)		535AP2, 587AP2
21	76/8.8/4.5	Hypothetical protein	KilA-N (pfam04383)	KilA-N domain-containing protein (*M. haemolytica*)		535AP2, 587AP2
22 *HNH*	168/19.4/9.6	HNH homing endonuclease	HNH_3 (pfam13392)	Putative HNH endonuclease (*Lactococcus*)		535AP2, 3927AP1, 1152AP2
23 *MTase*	198/21.3/8.3	Cytosine-specific DNA methyltransferease	Cyt_C5_DNA_methylase superfamily (cl18939)	Cytosine-specific methyltransferase (*Haemophilus parasuis*)		535AP2, 3927AP1, 1152AP2
24 *PK*	150/17.1−17.3/4.8−5	Pyruvate kinase		Hypothetical protein and pyruvate kinase (*M. haemolytica*)		
25 *exo*	211−225/23.5−25.9/ 4.8−5.9	Exonuclease	YqaJ (pfam09588)	Bacteriophage exonuclease (*M. haemolytica*)		
26 *bet*	264−307/29.2−35.3/5.2−5.3	Recombinase	RecT (pfam03837); bet_lambda (TIGR01913)	Bet protein (*M. haemolytica*)		
27	154/17.9/6.5	Hypothetical protein	NTP-PPase_u3 (cl16941)	Hypothetical protein (*M. haemolytica*)		
28 *higB*	91/10.8/6.9	HigB	HigB (COG3549)	Plasmid maintenance system killer (*M. haemolytica*)		587AP2
29 *higA*	101/11.4/8.1	HigA	Antidote_HigA (TIGR02607)	Plasmid maintenance system antidote protein (*M. haemolytica*)		587AP2
30 *TRase*	346/39.6/8.8	Transposase	HTH_28 (pfam13518)	Transposase (*M. haemolytica*)		587AP2
31 x*hlA*	161/18.5/5.5−5.8	Hypothetical protein	XhlA (pfam10779); TM (1)	Hypothetical protein (*M. haemolytica*)		
32 *cI*	219−228/24.8−26.3/4.9−5.5	CI repressor	S24_LexA-like (cd06529)	LexA family repressor/S24 family protease (*M. haemolytica*); bacteriophage transcriptional regulator (*Haemophilus parasuis*, for 587AP2)	20−24	
33 *cro*	68−90/7.5−10.1/6.1−9.1	Cro repressor	HTH_XRE (cd00093)	XRE family transcriptional regulator (*M. haemolytica*)	14−28	
34 *cII*	86/9.8/8	CII protein		Bacteriophage CII protein (*M. haemolytica*)	23	587AP2
35	80/9/9.6	Hypothetical protein	HTH_39 (pfam14090)	Hypothetical protein (*M. haemolytica*)		535AP2, 3927AP1, 1152AP2
36 *O*	276−289/31.8−33.3/8.9−9.1	Replication protein O	Phage_rep_O (pfam04492)	Bacteriophage replication protein (*M. haemolytica*)	18−21	587AP2
37 *P*	215/24.9/9.2	Replication protein P	Phage_lambda_P superfamily (cl06169)	Putative bacteriophage replication protein (*M. haemolytica*)	13	535AP2, 3927AP1, 1152AP2
38 *hel*	453/50.9/5.7	Helicase	DnaB (TIGR00665)	Replicative DNA helicase (*M. haemolytica*)		587AP2
39 *MTase*	178/20.7/8.8	Methyltransferase	MT-A70 (pfam05063)	Hypothetical protein and modification methylase MunIM (*M. haemolytica*)		587AP2
40 *MTase*	190/21.6/5	DNA N-6-adenine-methyltransferas	Dam superfamily (cl05442)	DNA N-6-adenine methyltransferase (*M. haemolytica*)		535AP2, 3927AP1, 1152AP2
41	150−168/17.7−19.9/6.1−8.6	Hypothetical protein	DUF1367 superfamily (cl06231)	Hypothetical protein (*M. haemolytica*)		
42 *ninG*	189/22.3/9.7	NinG protein	NinG (pfam05766)	Protein NinG (*M. haemolytica*)	43	587AP2
43	94/10.6/9	Hypothetical protein	DUF1364 (pfam07102)	Hypothetical protein (*M. haemolytica*)		535AP2, 3927AP1, 1152AP2
44 *RusA*	122/13.9/9.4	Endodeoxyribonuclease RusA	RusA (pfam05866)	Endodeoxyribonuclease RusA (*Haemophilus influenzae*)		535AP2, 3927AP1, 1152AP2
45 *Q*	121−157/14.3−18.6/9.2−9.5	Antitermination protein Q	Phage_antitermQ (pfam06530)	Phage anti termination protein Q (*M. haemolytica*)	18−22	
46 *S*	81−117/9.5−12.8/9−9.3	Holin	Phage_holin_3 superfamily;TM (1–3)	*Hemophilus*-specific protein (*M. haemolytica*)	11−17	
47 *R*	189−197/21.2−22.3/9.1−9.5	Endolysin	endolysin_autolysin (cd00737); lysozyme_like superfamily (cl00222); TM (1)	Lysozyme (*M. haemolytica*)	21−55	
48 *Rz*	116/13.2/7.8−8.6	Lytic protein Rz	DUF2570 (pfam10828); TM (1)	Hypothetical protein (*M. haemolytica*)	34−36	
49 *Rz1*	57−74/6.6−8.4/5−8	Lytic protein Rz1		Hypothetical protein (*M. haemolytica*)	30−44	

^a^TM: transmembrane α-helice; only 1152AP2 contains domain of Tape_meas_nterm superfamily (cl15680) and 587AP2 contains Phage_holin_3 superfamily; only protein J from 1152AP2 contains 2 TM

^b^Identity of amino acids sequence was calculated by ALIGN [66]

**Table 3:** Major gene products of *Mannheimia haemolytica* phage 3927AP2

CDS	Gene name	Size (aa)/MW(kDa)/pI	Motifs^a^	Function	% Identity (aa) to related Mu-like phages^b^	
					Su-Mu	phiMhaMu2
1	*c*	242/26.8/5.2	S24_LexA-like (cd06529)	Transcriptional regulatory protein	46.1	100
2	*ner*	75/8.4/9.8	HTH_Tnp_Mu_1 superfamily (cl15894)	DNA-binding protein	41.6	100
3	*A*	645/73.1/9.3	Mu-transpos_C (pfam09299)	Transposase	63.7	99.1
4	*B*	293/32.2/8.6	HTH_LacI (cd01392)	Mu-like prophage FluMu DNA transposition protein B	54.6	100
14	*gemA*	141/16.1/9.9	DUF1018 (pfam06252)	Mu-like prophage protein gp16	66	100
18	*mor*	141/16.0/7.5	Mor superfamily (cl02360)	Mor transcription activator family protein	70.9	100
19	*lys*	177/19.8/7.7	Glyco_hydro_108 (pfam05838) and PG_binding_3 (pfam09374)	Hypothetical protein	20	94.4
25	*DNA-binding*	166/18.4/6.1	TM (1)	DNA-binding protein	48.5	100
27	*terL*	541/62.3/6.2	Terminase_6 (pfam03237)	Terminase, large subunit	71.3	100
28	*porT*	541/59.9/5.2	COG4383 (COG4383)	Portal protein	84	100
29	*F*	434/50.1/9.3	Phage_Mu_F (pfam04233)	Phage head morphogenesis protein	73.6	100
30	*G*	138/15.3/5.8	COG5005 (COG5005)	Mu-like prophage FluMu G protein 2	73.9	100
31	*I*	356/38.9/5.2	Mu-like_Pro (pfam10123)	Bacteriophage Mu I protein Gp32	76.9	99.7
32	*T*	305/33.9/5.4	Mu-like_gpT (pfam10124)	Major head subunit T	75.6	100
34	*J/K*	141/15.9/4.7	DUF1320 (pfam07030)	Mu-like prophage protein gp36	71.5	100
35	*J/K*	213/23.6/5.9	DUF1834 (pfam08873)	Mu-like prophage protein gp37	69.5	100
37	*L*	469/50.7/5.7	Phage_sheath_1 (pfam04984)	Mu-like prophage FluMu tail sheath protein	74.1	85.1
38	*M/Y*	124/13.7/4.8	Tail_tube (pfam10618)	Phage tail tube protein	75.8	NA
41	*tmp*	759/81.5/9.2	PhageMin_Tail (pfam10145)	Tail length tape measure protein	64.2	100
42	*N*	430/48.6/5.4	DNA_circ_N (pfam07157)	Mu-like DNA circularization protein	45.8	100
43	*P*	375/41.8/7.6	Phage_GPD superfamily (cl15796)	Mu-like tail protein P	75	99.7
44	*Q*	219/22.8/6.9	Phage_Mu_Gp45 (pfam06890)	Mu-like baseplate assembly protein	70.8	99.5
45	*V*	116/13.1/6.8	GP46 (pfam07409)	Mu-like protein gp46	81.9	100
46	*W*	353/38.7/4.7	Baseplate_J superfamily (cl01294)	Mu-like baseplate J protein	54.1	100
47	*R*	196/21.9/5.2	DUF2313 (pfam10076)	Tail protein	35.8	99.5
48	*S*	686/75.7/5.9	Pectate_lyase_3 (pfam12708)	Mu-like prophage FluMu defective tail fiber protein	20.6	NA

^a^TM: transmembrane α-helice

^b^Identity of amino acids sequence was calculated by ALIGN [66]

### P2-like phages

**Fig. 2:**
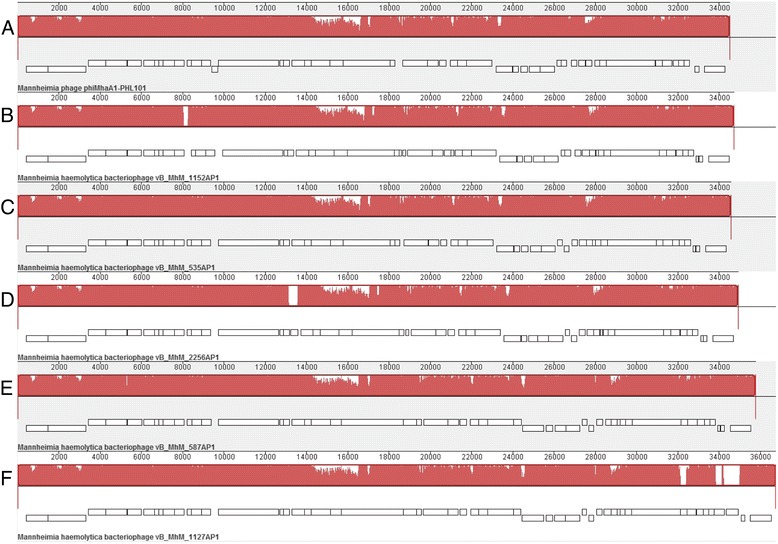
Whole genome comparisons of φMhaA1-PHL101 and all-P2 like *Mannheimia haemolytica* phages as well as using a progressive MAUVE alignment. The degree of sequence similarity is indicated by the intensity of the *red region*. The contiguous *black boxes* under the *red region* represent the position of genes. **a**, PHL101; **b**, 1152AP1; **c**, 535AP1; **d**, 2256AP1; **e**, 587AP1; **f**, 1127AP1

Phages 535AP1, 587AP1, 1127AP1 and 2256AP1 possess linear dsDNA of 34.9 to 35.7 kb in length with a G+C content of 41.5–42.1 %, encoding 51–53 CDSs (Additional file 1: Table S1, Additional file 2: Table S2, Additional file 3: Table S3, Additional file 4: Table S4). Comparative genomics revealed that 535AP1 and 2256AP1 are collinear with 98.5–98.7 % nucleotide similarity to P2-like phage φMhaA1-PHL101, previously identified within the genome of *M. haemolytica* [14], whereas 587AP1 and 1127AP1 exhibited 85.7 and 88.4 % similarity, respectively, to this phage (Fig. 2). Among the P2-like phages studied, including 152AP1 which we previously reported [13], genome 535AP1, 2256AP1 and 1152AP1 are more closely related (97.2–97.9 % pairwise identity), followed by 587AP1 and 1127AP1 with a similarity of 95.7 %. Computational analysis using CoreGenes [15–17] showed that 535AP1, 2256AP1, 587AP1 and 1127AP1 share 47 (95.9 %), 47 (95.9 %), 43 (87.8 %) and 42 (85.7 %) homologs with phage PHL101, respectively. Moreover, the tail fibre proteins of P2-like phages from serotype A1 (535AP1 and 2256AP1) and A6 (1152AP1) share an amino acid identity of >99 %, as did the tail fibre proteins from A2-induced phages (587AP1 and 1127AP1). But, the amino acid sequence of tail fibre proteins from A2-induced phages differe considerably (35 % ID) from those of either A1 or A6. Phage infection is generally initiated by specific adsorption to the bacterial cell surface. Bacterial receptors for phages belong to various biochemical families and are mainly represented by surface proteins, polysaccharides and lipopolysaccharides [18]. Well conserved tail fibres may reflect identical receptors shared within common or pathogenic *M. haemolytica* strains.

Similarly, a phylogenetic tree of structural genes (capsid and tail genes) of the known P2-like phages from different bacterial species reflected their respective host specificity [19]. To date, P2-like phages have been detected in a number of serotypes of *M. haemolytica* [9, 13, 14, 20], but differ from other members of the P2 genus [14], illustrating the specificity of temperate phages for their respective host [19].

### λ-like phages

**Fig. 3:**
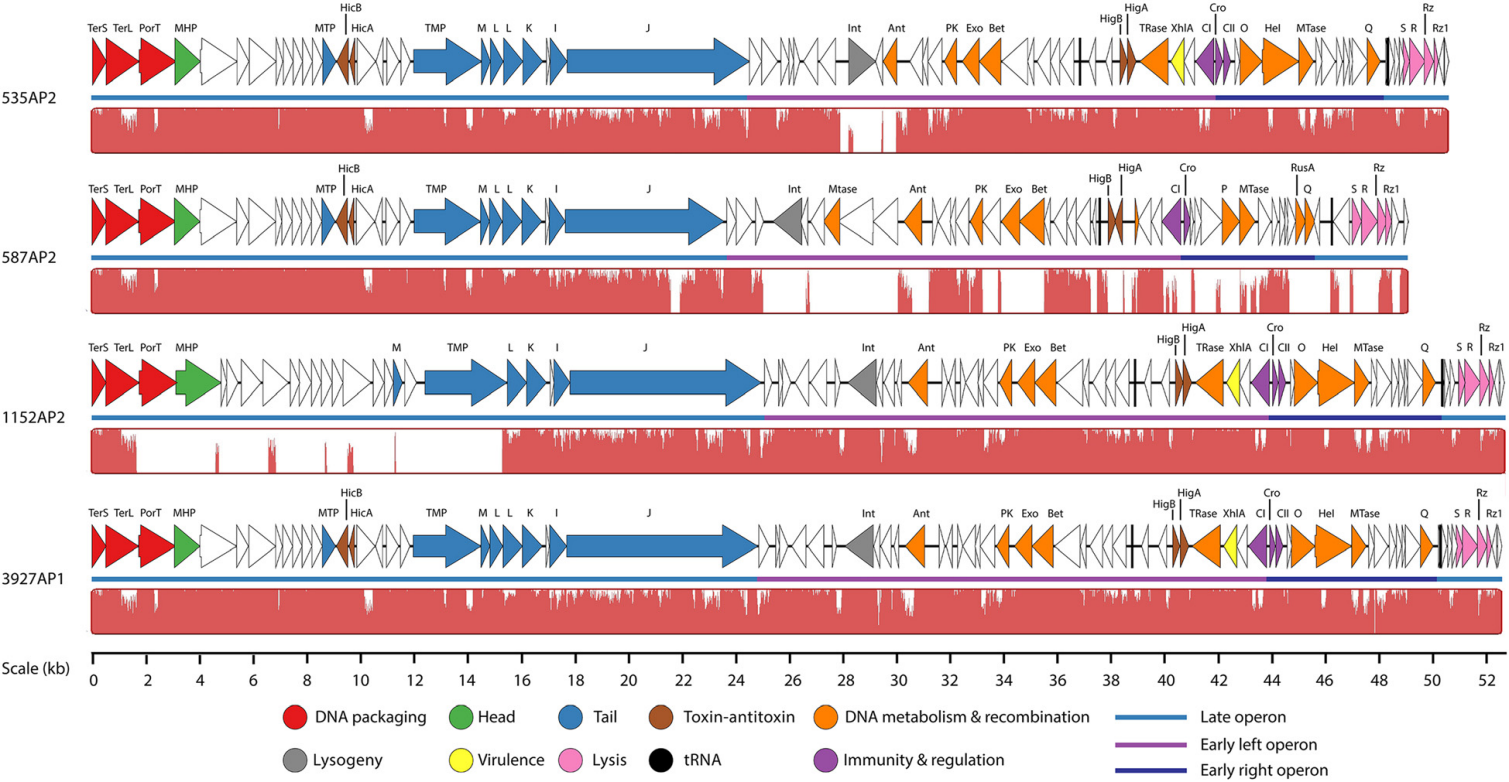
Genomic structure and nucleotide sequence comparison of λ-like *Mannheimia haemolytica* phages. Genetic map was constructed using Easyfig [67]. The four genomes were aligned using a progressive MAUVE alignment and the degree of sequence similarity is indicated by the intensity of the red region

The genomes of 535AP2, 587AP2, 1152AP2 and 3927AP1 range from 48.6 to 52.1 kb (41.1–41.4 % mol G+C) encoded for 77–83 CDSs and 2 tRNAs, and possessed a similar gene arrangement. Of the annotated gene products, 32–34 CDSs linked to essential functions which include components of the head and tail morphogenesis, infection specificity, site-specific recombination as well as replication initiation and cell lysis (Table 2 and Additional file 5: Table S5, Additional file 6: Table S6, Additional file 7: Table S7, Additional file 8: Table S8 and Fig. 3). Specifically, λ-like genes coding for minor tail proteins M, L and K, tail assembly protein I, host specificity protein J, integrase, replication proteins O and P, and antitermination protein Q were identified. However, these phage genome are not collinear with any other known λ-like phages, an outcome that was not unexpected as many prophages of the *Lambdalikevirus* from γ-proteobacteria maintain only the overall lambda-like synteny without demonstrating high sequence similarity [21]. To date, temperate phage Aaphi23 of *Actinobacillus actinomycetemcomitans* [22] is only λ-like phage with a complete genome sequence identified from the *Pasteurellaceae family*. Considering *A.actinomycetemcomitans* and *M. haemolyica* are both member of the *Pasteurellaceae and* their respective infecting λ-like phages *show* relatively high similarities at amino acid sequence level, we aligned the amino acid sequence of major gene products with known function of Aaphi23, respectively, with 535AP2, 587AP2, 1152AP2 and 3927AP. Irrespective of their function, amino acid sequences of the gene products from each λ-like phage studied exhibited low similarly (12–53 %) to those from Aaphi23 (Table 2), illustrating the highly mosaic nature of lambdoid phage genomes [21].

**Fig. 4:**
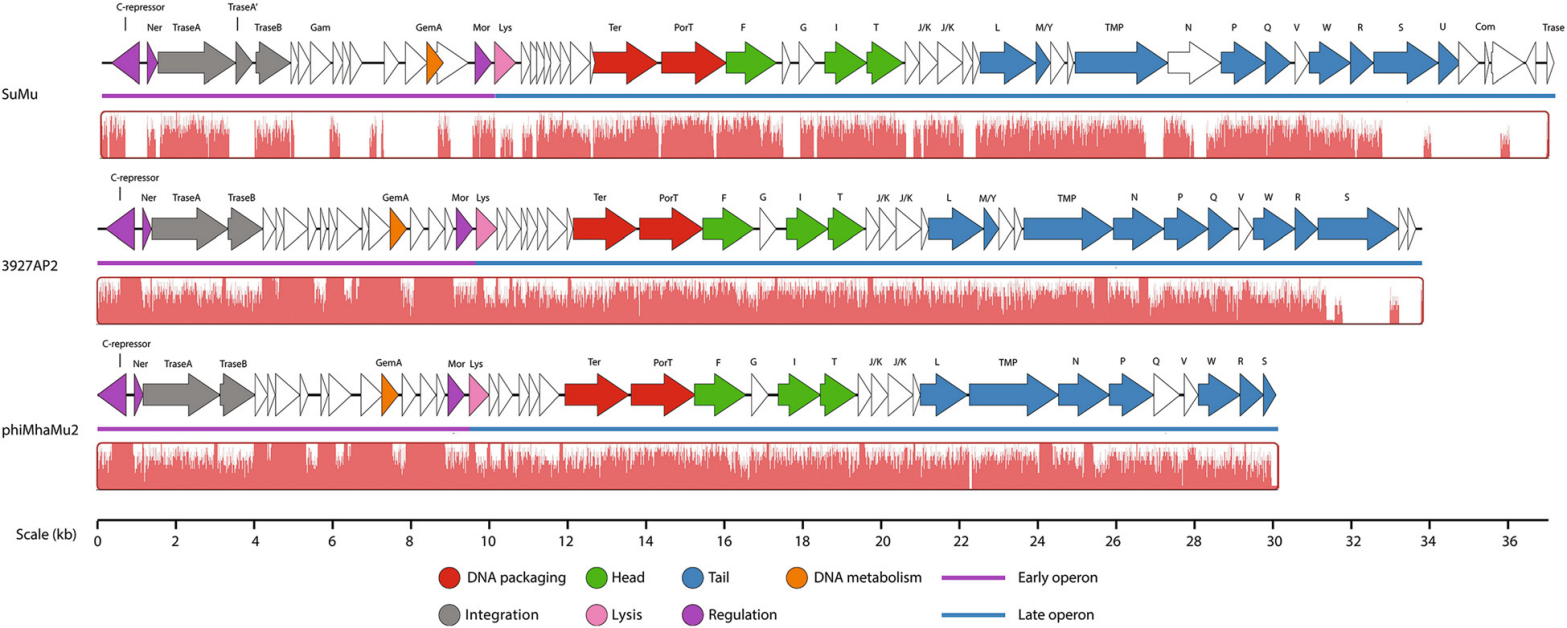
Genomic structure of *Mannheimia haemolytica* phage 3927AP2 and comparison with related Mu-like phages. Genetic map was constructed using Easyfig [67]. The four genomes were aligned using a progressive MAUVE alignment and the degree of sequence similarity is indicated by the intensity of the red region

Among the four λ-like phages, 535AP2 and 3927AP1 are the most genomically genetically similar (91.8 %), whereas phages 535AP2 and 3927AP1 are 77 and 84.9 % identical to 1152AP2, respectively (Fig. 4). The genome of 587AP2 diverges from the others exhibiting a pairwise sequence similarity of 72–73 % to 535AP2 and 3927AP1 and a low sequence identity of only 59.1 % to 1152AP2. Compared to the reference genome of 3927AP1 the majority of dissimilar regions were observed in 27.6-29.7 Kb of 535AP2, 24.8-48.2 Kb of 587AP2 and 1.7-15.2 Kb regions of 1152AP2 (Fig. 3). Likewise, comparative using GeneOrder4.0 [23] showed that 3927AP1 and 1152AP2 shared 72 (91.1 %) and 52 (65.8 %) of homologs with 535AP2. Phage 1152AP2 shares 62 (74.7 %) homologs in common with 3927AP1. Again, 587AP2 is very distant from the other three phages with 44–45 proteins in common with 535AP2 and 3927AP2 and only 26 homologs in common with 1152AP2. In addition, BLASTN showed that 97 % of a draft consensus of 2256AP2 is 99–100 % identical to 3927AP1 and 91 % of a draft consensus of 1127AP2 is 99 % identical to 587AP2 (data not shown), indicating that 2256AP2 and 1127AP2 are both likely lambdoid phages.

#### Integration and lysogenic control

A λ-like *int* gene was identified in the mid-region of the genome of all four phage (Table 2 and Fig. 3), which encodes integrase for the insertion of phage DNA into the bacterial chromosome [21]. Interestingly, *int gene* from 535AP2 is located in a cluster of genes transcribed is in the opposite orientation to the majority of the genes (Fig. 3), with a similar observation being made for the *int gene* of phage Aaphi23 [22]. Unlike typical lambdoid phages, these phages lack a *xis* gene coding for an excisionase upstream of *int* a situation also observed in lambiod phages Aaphi23 [22] and D3 from *Pseudomonas aeruginose* [24]. Others have observed that some staphylococcal prophages possess the *xis* gene for excision, while other prophage exclusively utilize integrase to excise from the host chromosome [25]. Further experiments are required to elucidate the excision mechanism among the four prophages associated with *M. haemolytica*. Between the *int* (CDS31) and the CDSs 42–44 promoting homologous recombination of 1152AP2, CDS33 contains a flap endonuclease-1-like domain (cl1485), CDS35 contains amino- and carboxy-domain of the phage P22 anti-repressor (cl11178 and cl11179), CDS36 contains a domain of LexA regulated protein (cl08198) and CDS37 contains a conserved DNA binding domain (pfam04383) (Table 2 and Additional file 7: Table S7). This suggests that this cluster of genes may be involved in regulating phage DNA integration and excision. A similar cassette of genes was also identified in 3927AP1, whereas 535AP2 and 587AP2 lack this gene cluster.

#### Immunity and regulation 

A typical λ-like gene cassette of *cI−cro* was identified in all λ-like phages (Table 2 and Fig. 3). Phages 535AP2, 1152AP2 and 3927AP1 share an identical repressor protein CI (100 % aa ID). Moreover, of 91 amino acid sequences of repressor Cro protein from 535AP2, 69 are perfectly aligned with that at N-terminus from 1152AP2 and 3927AP2. In contrast, CI (CDS56) and Cro (CDS57) proteins of 587AP differ remarkably (less than 21 % aa ID) from their counterparts in the other three phages. The CI and Cro regulators maintain the lysogenic and lytic states, respectively, as a bistable genetic switch [26]. The CI is able to repress Cro and vice versa. After infection of a target bacterium, the decision between lytic or lysogenic development of phage lambda is based upon environmental signals and the number of infecting phages per cell. Additionally, the prophage may enter lytic development in response to DNA-damaging agents. Noticeably, unlike 535AP2, 1152AP2 and 3927AP1; 587AP2 lacks *cII* for stimulating CI transcription, although the CDS59 of 587AP2 was predicted to contain an HTH motif, suggestive of the presence of a CII homolog. The immunity and regulation of the phages induced from *M. haemolytica* serotype A1 and A6 differ from those induced from *M. haemolytica* serotype A2, indicating that λ-like phages may employ different mechanisms in regulating their life circle between common and pathogenic serotypes of *M. haemolytica*.

#### Host recognition

All four λ-like phage encod a host specificity tail protein J (also called tail fiber) ranging from 1955 to 2353 amino acids in length, which functions to bind to the host receptor. Protein J id only well conserved between 1152AP2 and 3927AP1 (98.7 % aa ID), especially at the C-terminus, although all four phages exhibited an amino acid sequence identity of >86.5 % for this protein. Phages 1152AP2 and 3927AP1 were both induced from *M. haemolytica* A6 and shares an identical host recognition protein, confirming their origin from a common host.

#### Host cell lysis

Genes coding for holin, endolysin, lysis proteins Rz and Rz1 were identified from the right end of the genome of all four λ-like phages (CDSs 72–81, Table 2, Additional file 5: Table S5, Additional file 6: Table S6, Additional file 7: Table S7, Additional file 8: Table S8, Fig. 3) which are organized similarly to the archetypical lambda lysis cassette *SRRzRz1* [21]. Holins are small transmembrane proteins that form non-specific pores in the bacterial cytoplasmic membrane for exporting endolysins to the bacterial cell wall [27]. Based on a new classification scheme of holins as proposed by Reddy and Saier Jr. [27], all known holins can be divided into 7 superfamilies (I–VII). All members of Superfamily II contain 78 ± 14 amino acids and are predicted to have 1 to 2 transmembrane α-helices (TMs). The CDS74 of phages 535AP2 and 1152AP2 as well as CDS78 of 3927AP1 (81aa) located immediately upstream of the endolysin gene, did not align with any homologs with holin function. However, they were predicted to have 1 TMD, a dual-translation start regulatory motif (MKLM) [28] and a highly charged, hydrophilic, C-terminal domain. Collectively these features indicate that these CDS are likely members of Superfamily II holin.

#### Virulence encoding genes

A gene product of 162 amino acids was predicted to have 1 TM and a XhlA (pfam10779) domain in the early left operon of each phage (Table 2). XhlA is a cell-surface associated haemolysin that lyses granulocytes and plasmatocytes immune cells of insects [29]. Cowles et al. [29] demonstrated that XhlA is required for full virulence of the γ-proteobacterium *Xenorhabdus nematophila*, towards *Manduca sexta* larvae. In addition, XhlA shows haemolytic activity against mammalian erythrocytes in vitro [29]. To date, there is no report of haemolysin XhlA playing a role in *M. haemolytica* infection and further experimentation is required to verify whether this phage-encoded protein plays a role in *M. haemolytica* pathogenesis.

#### Toxin-antitoxin gene cassettes

All λ-like phages contain genes encoding for a toxin-antitoxin (TA) system (Table 2). Prophages 535AP2 and 3927AP1 encod 2 TA, located in early (*higBA*) and late operon (*hicAB*), respectively, whereas 1152AP2 encod 1 TA (*higBA*) in the early operon and 587AP encod 1 TA (*hicAB*) in the late operon. The first TA cassettes were characterized as plasmid-borne ‘killer’ genes that ensure plasmid maintenance after cell replication by eliminating plasmid-free cells [30]. However, TA systems are not only restricted to plasmids, but have also been identified in the chromosome of bacteria and archaea, where they function to regulate bacterial programmed cell death, biofilm formation, cope with nutritional stress, establish persister subpopulations and offer protection from phage attack [31]. As well, the TA systems have been identified in *E. coli* temperate phage P1, N15 and streptococcal temperate phage [32–34]. In the temperature sensitive plasmid Rts1 from *E. coli* K12, the *higBA* locus encodes HigB toxin and HigA antitoxin, which stabilize plasmid Rts1 by inhibiting the growth of plasmid-free cells [30]. The *hicAB* locus of *E. coli* K12 encodes HicA and HicB, which help the cell cope with nutritional stress [35]. Presumably, the *higBA* and/or *hicAB* pairs may act on the toxin-antitoxin principle to stabilize inheritance of the four prophages within their host chromosome, as previously observed of the *phd*/*doc* cassettes in phage P1 [34].

#### Transposase

Excluding 587AP2, transposase-encoding genes were discovered immediately downstream of the XhlA domain in phages 535AP2, 1152AP2 and 3927AP1 (Table 2, Additional file 5: Table S5, Additional file 7: Table S7 and Additional file 8: Table S8, Fig. 3). Transposases were also identified from P2-like phages of 587AP1 (CDS31), 1127AP1 (CDSs 31 and 50) and 2256AP1 (CDS17) (Additional file 1: Table S1, Additional file 2: Table S2, Additional file 3: Table S3, Additional file 4: Table S4). Transposases are responsible for catalysing relocation, transposition and horizontal transfer of mobile genetic elements such as transposon within and/or between genomes [36]. A well characterized transposase MuA, is required for insertion of phage Mu genome into the host chromosome as well as replication of the phage DNA during the lytic cycle [37]. Transposase genes have also been detected within the genomes of *Staphylococcus* lytic phages [38, 39] and P2-like phages of *Burkholderia cepacia* [40]. Existence of the transposase-encoding genes in the λ-like phages and P2-like phages studied, suggests that they play a role in the acquisition of foreign genes from other bacteria or other phages.

#### Methyltransferease

A methyltransferease coding gene was identified upstream of *int* in 535AP2 (CDS37, pfam13649) and 587AP2 (CDS34, cytosine-specific DNA methyltransferase, cl18939) (Table 2, Additional file 5: Table S5 and Additional file 6: Table S6, Fig. 3). Another methyltransferase was identified immediately downstream of helicase coding genes of all the λ-like phages (Table 2 and Additional file 5: Table S5, Additional file 6: Table S6, Additional file 7: Table S7, Additional file 8: Table S8, Fig. 3). Specifically for 587AP2, a DNA N-6-adenine-methyltransferase (*Dam*, cl05442) was identified. Methyltransferase functions as a powerful epigenetic gene regulator switching genes on and off by adding a methyl group to a particular base within a defined short DNA sequence. The enzyme is frequently found in various prokaryotic and eukaryotic cells [41, 42] and plays a pivotal role in regulating virulence genes as well as repairing mismatches during DNA replication in bacteria [42]. Although methyltransferase is commonly found in phages, its function remains unclear. Methyltransferase may play a role in regulating the life cycle of phages, confer protection against host restriction systems and modify the expression of virulence genes in the host [42, 43].

### Mu-like phages

Mu-like phage 3927AP2 consists of 33.8 kb of double strand genome (43.1 % mol G+C) in length, encoding 50 CDSs (Tables 1, 3 and Additional file 9: Table S9; Fig. 4). Comparative genomic analysis showed that this phage is 88.8 % identical to prophage Mu remnant phiMhaMu2 present in PHL213 strain of *M. haemolytica* [9], but only 47−59 % similar to *Heamophilus parasius*-infecting phage SuMu (59 % similarity) [44] as well as other known Mu-like phages [45]. Of 50 CDSs, 26 resemble functions within Mu-like phages including DNA metabolism and packaging, immunity and regulation, head and tail structures as well as lysis function (Table 3 and Fig. 4). Moreover, the amino acid sequence of these Mu-like gene products in 3927AP2 better align with those from phiMhaMu2 (85–100 % ID) than from SuMu (20.6–84 % ID) (Table 3). Specially, extreme low identity (20–21 %) of amino acid sequence between 3927AP2 and SuMu occur in genes of *lys* and *S*, which encode endolysin and tail fibres, respectively. *M. haemolytica* and *H. parasius* both belong to the *Pasteurellaceae* family, but their respective infecting Mu-like phages differ considerably in genes for host recognition and lysis, confiming the intragene mosaicism of Mu-like phages [45]. High DNA nucleotide and amino acid sequence similarity shared between 3927AP2 and phiMhaMu2 suggest that Mu type phages of *M. haemolytica* may possibly be more related.

Typically, the Mu-like phage module is divided into early, middle and late regions on the basis of the level of transcription at different times during the lytic phase of Mu’s life cycle [45]. Comparing with phage Mu [45], diverse genes were more identified from the early region (12/18, 67 %) than from the late region (13/32, 41 %) in 3927AP2. This suggests that early and middle regions are less conserved than the late regions in Mu type phages [45]. In phage Mu [46], the semi-essential early (SEE) region located between the *B and C* genes (4.3–10 kb) contains *kil, gam, sot, arm, cim* and *gemA/B* (the *gemB* also known as *mor*), which are involved in DNA replication, immunity and regulation as well as host killing function. In contrast, only *gam* and/or *gemA/B* were annotated in SEE region of phages 3927AP2, phiMhaMu2 and SuMu. Presumably, other hypothetical proteins located in this region are responsible for the functions described above and/or some of these SEE genes may be lost depending upon selective pressures and a lack of their necessity for phage development [45]. Another striking feature shared among phages 3927AP2, phiMhaMu2 and SuMu is that the late gene of *lys is* located immediately downstream of the early genes of *mor*, indicating that these phages lack middle genes such as *C*, a transcription activator for late genes transcription [46]. Also, no typical *com-mom* module was identified at the right extremity of the 3927AP2 genome. A pair of *com-mom* genes is utilized for phage Mu to regulate late gene transcription and expression [46]. As a consequence, experimental investigation is required for identification of genes encoding similar function to C, Com and Mom.

### Inducible prophages

Together with the P2- and λ-like phages induced, PHAST [12] analysis detected more intact prophages including Mu-like phages in the genomes of 535A and 587A (Data not shown). In contrast, the genome of 3927A only carried λ- and Mu-like phages. Interestingly, Mu-like phages were only induced from 3927A and not from 535A or 587A. It is unclear as to why Mu-like phages were not induced in 535A or 587A even though they were clearly present in the genome of these strains. It may be related to the method of induction, or possibly competitive interactions among multiple prophages occurring within the host during induction using mitomycin C. When P2-, λ- and Mu-type prophages co-exist in *M. haemolytica* chromosome, the former two may be more sensitive to switching to the lytic state or have the capability of out-competing the latter for the resources required for DNA replication within the host cell. The implications of within-host competition between co-infecting phages are largely undefined. However, Refardt [47] studied within-host competition between lambdoid phages induced using mitomycin C with *E. coli*. Singularly, both phages were equally inducible, but when combined replication of one of the phage was highly restricted. Likewise, James et al. [48] reported that an inducible siphovirus LESφ2 outcompeted two co-infecting siphorviruses when norfloxacin was used to induce phages from *Pseudomonas aeruginosa* strain LESB58. We also cannot exclude the possibility that some of the intact phages within the genome of *M. haemolytica* may no longer be active or inducible. In *Lactobacillus plantarum* strain WCFS1, prophages Lp1 and Lp2 with genome size of 40 kb as members of Sfi11-like, *Siphoviridae*, are not inducible using mitomycin C [49]. Prophages seem to be only transient passengers of the bacterial chromosomes, with some decaying and eventually being lost from the genome. However, even with this evolutionary process occurring, up to 20 % of bacterial genome can be accounted for by phage and their associated genes [6]. Inducible prophages may be of greater significance than uninducible prophages as they can play a role in horizontal gene transfer and disseminate virulence determinants and other genetic traits among bacteria [48, 50]. One may argue that carriage of phages that are prone to induction may represent a significant burden to host cells, selecting against their persistence in natural populations. However, P2- and λ-type prophages have been widely reported with the *M. haemolytica* genome [8, 9, 13, 51].

### Endolysin

According to CLUSTAL multiple sequence alignment results, four types of endolysins including P2-like, λ-like, 587AP2-like and Mu-like were discovered in this study. Additional file 10: Figure S1 illustrates the tertiary structure of the lysins with 87 to 92 % residues modelled with 100 % confidence. The P2-like endolysins contain 188 amino acids and are virtually identical (pairwise sequence identities, 99 to 100 %) at both the nucleotide and amino acid level. Likewise, endolysins from λ-like phages 535AP2, 1152AP2 and 3927AP1 are composed of 189 amino acids with identical nucleotide sequences. Interestingly, the genome of 587AP2 did not align with λ-like endolysins, exhibiting low nucleotide (46.8 %) and amino acids identities (19.2 %). Endolysins are hydrolases produced by phages to degrade the peptidoglycan layer of the bacterial cell wall, enabling release of phage progeny. Their antibacterial activity is highly specific and in situ application of endolysins has been shown to reduce bacterial colonization in the respiratory and vaginal tract of mice and humans [52]. Previously, application of endolysins against gram negative bacteria was limited as their outer membrane blocks their access to the peptidoglycan layer. Recent developments have overcome this limitation by combining endolysins with peptides that disrupt the outer membrane [53]. Thus, with this approach endolysin-based products may be developed that have activity against *M. haemolytica* and aide in the prevention of BRD.

## Conclusions

P2-, λ- and Mu-like phages were simultaneously induced from individual *M. haemolytica* isolates. Moreover, when these three types of phages co-existed within the *M. haemolytica* genome, P2- and λ-like phages were only recovered after induction, suggesting that within-host competition might exist among P2-, λ- and Mu-like phages with Mu-like phages being less competitive for lytic resources. Toxin-antitoxin gene cassettes in λ-like phages suggest that these genetic elements may contribute to the development of persister subpopulations of *M. haemolytica*. Cell-associated haemolysin XhlA encoded within λ-like phage genomes suggests that this element may contribute to the pathogenicity of *M. haemolytica*. Further investigations are required to verify how phages contribute to the pathogenesis and ecological fitness of *M. haemolytica*.

## Methods

### Temperate phages induction

Six field *M. haemolytica* isolates representing serotypes A1 (*n* = 2; 535A, 2256A), A2 (*n* = 2; 587A, 1127A) and A6 (*n* = 2; 1152A, 3927A) were selected for induction of temperate phage, as described previously [13]. Bacterial isolates were collected from healthy cattle housed in two commercial feedlots in Alberta, Canada. Phage filtrates were stored at 4 °C prior to DNA extraction.

### Induction growth curve

To plot the growth of *M. haemolytica*, with and without mitomycin, overnight cultures of each strain (*n* = 6) were diluted (1:10) in brain-heard infusion broth (BHI) and replicates (*n* = 14) were incubated in 96-well microplates at 37 °C. Optical density at 600 nm was recorded every 15 min to measure growth. When the absorbance of each strain reached log-phase (OD600 = 0.25–0.3), mitomycin C (Sigma Aldrich Canada Ltd., Oakville, ON; 10 ng/ml) was added to a final concentration of 0.2 μg/ml in half of the wells (*n* = 7), while the other wells received a similar volume of sterile water (*n* = 7). After induction with mitomycin C, absorbance at 450 and 600 nm was recorded at 15 min intervals for 12 h. Absorbance measures at 600 nm were subtracted from the 450 nm values, to give a final corrected optical density.

### Genome sequencing and annotation

Phage genomic DNA was isolated from each single induced preparation. Bacterial nucleotides were removed from the six filtered phage lysates using DNase 1 (Sigma-Aldrich) and RNase A (Sigma-Aldrich), and the phage lysates were concentrated using polyethylene glycol (PEG) 8000 [54]. Genomic phage DNA was extracted from concentrated phage suspensions using proteinase K (Qiagen, Toronto, ON) and a Phage DNA Isolation Kit (Norgen Biotek Corp., Thorold, ON) according to manufacturer’s instructions. Extracted DNA was quantified fluorometrically using the Quant-iT PicoGreen dsDNA Assay Kit (Invitrogen, Burlington, ON) on a NanoDrop 3300 fluorospectrometer (Fisher Scientific Limited, Nepean, ON). Subsequent DNA quality control assurance and amplification of the six phage samples was conducted by Eurofins MWG Operon (Huntsville, AL) prior to sequencing by GS FLX Titanium series chemistry (Roche 454). Whole genome sequencing yielded 100 to 300× coverage. Sequencing data were assembled by Celera Assembler (Version 5.3) and Staden gap4 and critical gaps were identified and closed by conventional Sanger sequencing. Whole genome sequence data of *M. haemolytic* isolates 535A, 587A and 3927A were also used for confirmation of the assembly of phage genomes and to identify phage genomes that were within the bacterial genomes, but not recovered by induction (Data not shown). Initial genome annotation was completed using myRAST [55]. SeqBuilder application (DNASTAR, Inc., Madison, WI) was used to visually scan the sequence for potential genes. All translated proteins were scanned for homologs using BLASTP and PSI-BLAST [56]. Rho-independent terminators were identified using WebGeSTer at http://pallab.serc.iisc.ernet.in/gester/rungester.html [57] and TransTermHP [58]. Promoters were identified by neural network promoter prediction [59] along with manual annotation. Transmembrane domains were described using TMHMM 2.0 at http://www.cbs.dtu.dk/services/TMHMM/ [60], Phobius at http://phobius.sbc.su.se/ [61] and SPLIT 4.0 at http://split.pmfst.hr/split/4/ [62]. Pairwise nucleotide sequence identity was calculated by EMBOSS Stretcher analysis [63, 64]. CLUSTAL omega [65] was used to align amino acid sequences of tail fibres proteins and endolysin proteins. ALIGN [66] at http://xylian.igh.cnrs.fr/bin/align-guess.cgi was used to generate amino acid identities of gene products. The GenBank accession number for 535AP1, 535AP2, 587AP1, 587AP2, 1127AP1, 1152AP2, 2256AP1, 3927AP1 and 3927AP2 sequences are KP137432, KP137433, KP137434, KP137435, KP137436, KP137437, KP137438, KP137439 and KP137440, respectively.

## Additional files

Additional file 1: Table S1. Feature of phage 535AP1 gene products and their functional assignments. (XLSX 13 kb) Additional file 2: Table S2. Feature of phage 587AP1 gene products and their functional assignments. (XLSX 13 kb) Additional file 3: Table S3. Feature of phage 1127AP1 gene products and their functional assignments. (XLSX 13 kb) Additional file 4: Table S4. Feature of phage 2256AP1 gene products and their functional assignments. (XLSX 13 kb) Additional file 5: Table S5. Feature of phage 535AP2 gene products and their functional assignments. (XLSX 21 kb) Additional file 6: Table S6. Feature of phage 587AP2 gene products and their functional assignments. (XLSX 15 kb) Additional file 7: Table S7. Feature of phage 1152AP2 gene products and their functional assignments. (XLSX 17 kb) Additional file 8: Table S8. Feature of phage 3927AP1 gene products and their functional assignments. (XLSX 16 kb) Additional file 9: Table S9. Feature of phage 3927AP2 gene products and their functional assignments. (XLSX 14 kb) Additional file 10: Figure S1. Teritiary structure of four types of lysins (**a**, P2-like; **b**, λ-like; **c**, 587AP2; **d**, Mu-like) with 87 to 92 % residues modelled with 100 % confidence generated by Phyre V2.0 [68] (http://www.sbg.bio.ic.ac.uk/phyre2/html/page.cgi?id=index). Image coloured by rainbow N → C terminus, model dimensions (Å):X:53.821; Y:51.063; Z:34.680. (TIFF 948 kb)

## Abbreviations

BLASTN: Basic local alignment search tool-nucleotide
BRD: Bovine respiratory disease
CDS: Coding sequence
DNA: Deoxyribonucleic acid
G+C: Guanine plus cytosine
int gene: Intergrase gene
PHAST: Phage search tool
TA: Toxin-antitoxin
TM: Transmembrane α-helices
tRNA: Transfer ribonucleic acid
XhlA: Cell associated haemolysin
xis: Gene coding for an excisionase
