# Supplementary figures and images for: Comparative analysis of multiple inducible phages from Mannheimia haemolytica
Source: BMC Microbiol. 2015 Aug 30;15:175. doi: 10.1186/s12866-015-0494-5 (PMC4553209; doi:10.1186/s12866-015-0494-5)

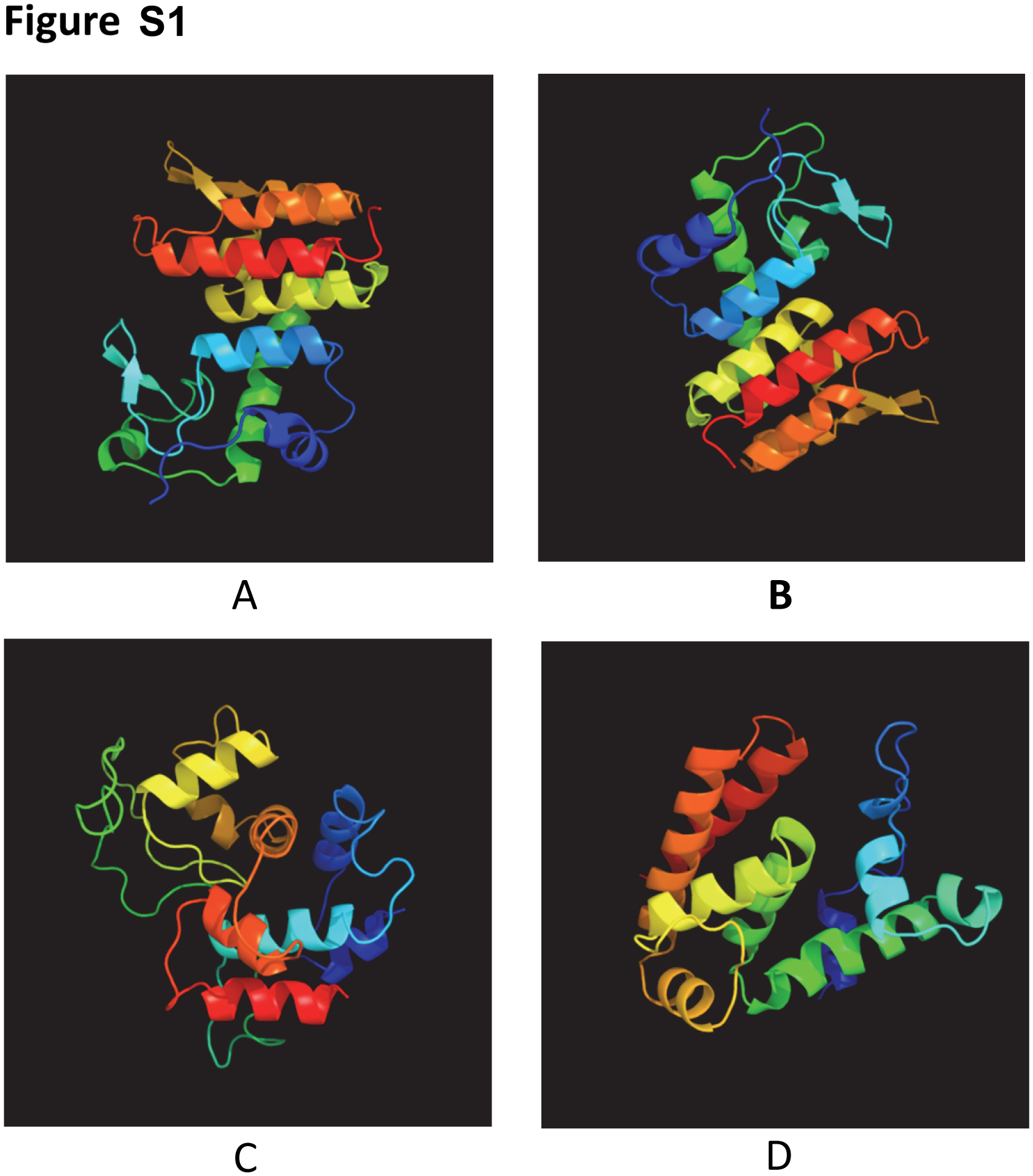

Supplement: Additional file 10: Figure S1. — Teritiary structure of four types of lysins (a, P2-like; b, λ-like; c, 587AP2; d, Mu-like) with 87 to 92 % residues modelled with 100 % confidence generated by Phyre V2.0 [68] (http://www.sbg.bio.ic.ac.uk/phyre2/html/page.cgi?id=index). Image coloured by rainbow N → C terminus, model dimensions (Å):X:53.821; Y:51.063; Z:34.680. (TIFF 948 kb) [file 12866_2015_494_MOESM10_ESM.tiff]
